# Spatial And Temporal Trends Of Organic Pollutants In Vegetation From Remote And Rural Areas

**DOI:** 10.1038/srep25446

**Published:** 2016-05-05

**Authors:** Mireia Bartrons, Jordi Catalan, Josep Penuelas

**Affiliations:** 1 CSIC, Global Ecology Unit CREAF-CSIC-UAB. Cerdanyola del Vallès 08193, Barcelona, Catalonia, Spain; 2 BETA Technological Centre (Tecnio), Aquatic Ecology Group, University of Vic–Central University of Catalonia. Vic 08500, Barcelona, Catalonia, Spain; 3 CREAF. Cerdanyola del Vallès 08193, Barcelona, Catalonia, Spain

## Abstract

Persistent organic pollutants (POPs) and polycyclic aromatic hydrocarbons (PAHs) used in agricultural, industrial, and domestic applications are widely distributed and bioaccumulate in food webs, causing adverse effects to the biosphere. A review of published data for 1977–2015 for a wide range of vegetation around the globe indicates an extensive load of pollutants in vegetation. On a global perspective, the accumulation of POPs and PAHs in vegetation depends on the industrialization history across continents and distance to emission sources, beyond organism type and climatic variables. International regulations initially reduced the concentrations of POPs in vegetation in rural areas, but concentrations of HCB, HCHs, and DDTs at remote sites did not decrease or even increased over time, pointing to a remobilization of POPs from source areas to remote sites. The concentrations of compounds currently in use, PBDEs and PAHs, are still increasing in vegetation. Differential congener specific accumulation is mostly determined by continent—in accordance to the different regulations of HCHs, PCBs and PBDEs in different countries—and by plant type (PAHs). These results support a concerning general accumulation of toxic pollutants in most ecosystems of the globe that for some compounds is still far from being mitigated in the near future.

Hundreds of thousands of organic compounds produced synthetically for use in agricultural, industrial, and domestic applications have been annually spread widely around the globe since the beginning of the industrial revolution^1^. Some of these compounds known as persistent organic pollutants (POPs) are intentionally produced for use as pesticides (e.g. hexachlorobenzene (HCB), also a byproduct in the manufacture of chlorinated solvents; hexachlorocyclohexanes (HCHs); and dichlorodiphenyltrichloroethanes (DDTs)), industrial fluids (polychlorinated biphenyls (PCBs)), and flame retardants (polybromodiphenyl ethers (PBDEs)) (Table S1). Others, such as polycyclic aromatic hydrocarbons (PAHs) are unintentional byproducts of the incomplete combustion of organic matter. POPs and PAHs are toxic and semivolatile and have the capacity to bioaccumulate and biomagnify in food webs. These chemicals enter food webs predominantly through the equilibrium partition of primary producers with the atmosphere^2^. In terrestrial ecosystems, the accumulation of POPs in vegetation is thus the major vector of POPs and, consequently, a crucial process in determining the extent of environment (and human) exposure to POPs. High concentrations of POPs and PAHs can indeed cause endocrine disruption, altered neurological development, immune system modulation, and cancer^3^.

The atmosphere is the principal source of POPs and PAHs in vegetation^4^,^5^,^6^. POPs and PAHs enter vegetation by gaseous equilibrium partitioning between the vegetation and surrounding air and by dry/wet particle-bound deposition; partitioning from soil to plant roots is a secondary entrance pathway. In contrast to hydrophilic compounds^7^, lipophilic pollutants such as POPs and PAHs are generally not translocated within a plant through the phloem, because they are retained in lipophilic tissues, and thus the metabolism of the compounds is not significant^8^. All these mechanisms of pollutant uptake and bioaccumulation in plants are mostly dependent on the physicochemical properties of the compounds but also on environmental (e.g. temperature, soil composition, and wind velocity) and especially on plant (e.g. surface area, lipid and VOCs content—, hydration state and growth rate) parameters^9^,^10^,^11^. However, some researchers hold that the equilibrium between plants and air is slow and thus plant tissues accumulate POPs throughout their life span, which reinforces the role of plant physiological adaptations and life cycle in the bioaccumulation of POPs^10^,^11^.Anyway, the highest theoretical maximum reservoir capacity for POPs is found in areas with low temperatures and coniferous forests (e.g. Siberia, Canada, and Scandinavia) where both the gas-to-leaf partition and the plant characteristics such as surface area are maximized, and the lowest concentrations are typically in warm and desert areas (e.g. Sahara) where most semivolatile compounds are in the gas phase and organic matrices able to retain POPs are rare^12^. Compounds retained in leaves by this filtering effect ultimately accumulate in soils under forest canopies after leaf senescence because they have reduced mobility across the phloem. Soils under forest canopies accumulate 3–5 times more POPs than bare soils^13^, and this retention substantially increases their overall global residence times^14^.

The widespread distribution and environmental and health concerns of POPs and PAHs led to international environmental agreements to eliminate or restrict their production, use, and release. The first global regulation to outlaw, limit the use or curtail inadvertent production of POPs was agreed in the Stockholm Convention on Persistent Organic Pollutants in May 2001 that entered into force on May 2004. Twelve groups of substances, informally known as the *dirty dozen*, were initially included. Little information is available for the temporal trends of these compounds in ecosystems, but a decrease in POP dispersion has been detected since the onset of the regulations^15^,^16^. POP concentrations, however, are still high in most ecosystems, causing health risks to humans and wildlife^17^,^18^, and substantial gaps in information remain about the temporal trends of POPs in ecosystems and the levels of toxicity and environmental stress incurred at the level of the ecosystem. A wide range of POPs has been remobilized into the atmosphere of the remote Arctic over the past two decades as a result of climate change, confirming that Arctic warming could undermine global efforts to reduce environmental and human exposure to these toxic chemicals^19^ and underscoring the need for an assessment of current temporal and spatial trends of POPs.

We examined the trends of temporal and spatial accumulation of airborne organic contaminants in vegetation at a global scale from an ecosystem perspective (i) to assess the level of contamination by POPs and PAHs in a maximized number of plant species—as the first entrance of POPs in food webs and thus crucial in determining the extent of wildlife and human exposure to POPs; (ii) to assess the main factors affecting the concentrations of POPS and PAHs in the different ecosystems; (iii) to assess the impacts of international POP regulations on these concentrations; and verify the limited available data and models indicating that atmospheric levels of most POPs are declining slowly in some regions of the northern hemisphere^16^,^20^. The target analytes included current-use (DDTs – only for malaria and dengue control – PBDEs and PAHs) and historic-use (HCB, HCHs, and PCBs) pollutants in vegetation from industrial, urban, rural and remote areas.

## Results

**Figure 1:**
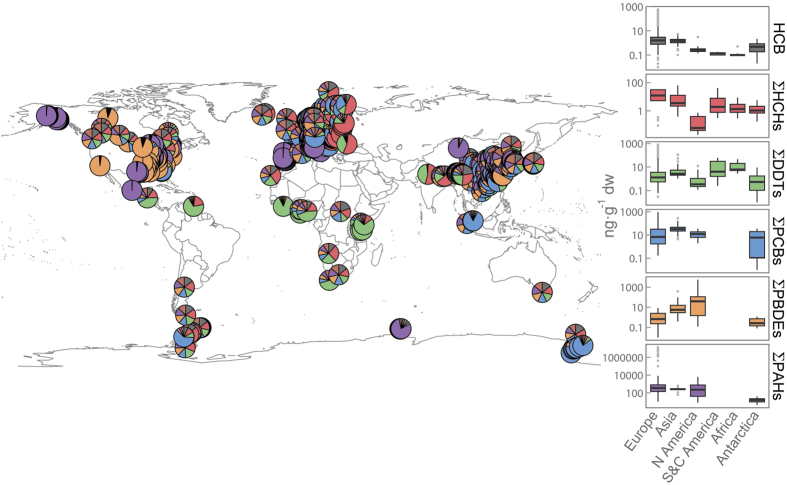
Continental distribution of POPs in vegetation around the globe. The map shows the proportion of each family of compounds reviewed for all sampling locations with published data. The boxplots compare the concentrations of each family of POPs across continents. Interquartile ranges (25^th^ and 75^th^ percentile) are shown by the height of the boxes, and the horizontal lines represent medians (50^th^ percentile). Whiskers range from the 10^th^ to 90^th^ percentiles. Concentrations are in ng·g^−1^ dry weight. The map was generated using the R packages *rgdal*^60^*, maptools*^61^, and *mapplots*^62^. Permission is granted to Nature Publishing Group to publish the image in all formats under an Open Access license.

We found 79 publications providing concentrations of HCB, HCHs, DDTs, PCBs, PBDEs, and PAHs in vascular plants, mosses and lichens from around the globe (mean values and references in Table S2). The publications spanned 1977 to 2015 and 54% of the studies included rural areas (Fig. S1). The Northern Hemisphere, particularly Europe, was best represented (Fig. 1), but data from South and Central America, Africa, and Australia were limited: 1650 observations in Europe versus 204 in North America, 15 in South and Central America, 28 in Africa, 427 in Asia, and 235 in Antarctica. Most of the studies focused on gymnosperms, particularly *Pinus sylvestris* (388 of 727 observations), and information on bryophytes, lichens, and angiosperms were lacking (Table S2).

**Figure 2:**
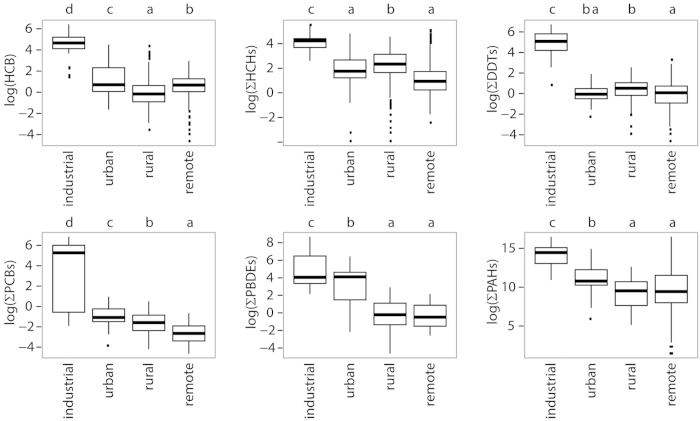
Variability of POPs and PAHs in vegetation from remote, rural, urban, and industrial environments. Box plots of the logarithm of the concentration of each family of compounds. Small letters refer to significant differences among land uses (Tukey’s HSD, *P* < 0.05). Different letters indicate significant differences. Interquartile ranges (25^th^ and 75^th^ percentile) are shown by the height of the boxes, and horizontal lines represent medians (50^th^ percentile). Whiskers range from the 10^th^ to 90^th^ percentiles, and values outside this range are indicated by small squares. Concentrations are in ng·g^−1^ dry weight.

Concentrations of POPs and PAHs were high on most continents (Fig. 1), particularly Asia, Europe, and North America for most compounds; and Antarctica usually had the lowest concentrations. Central and South America, Africa, and Asia had the highest p,p′-DDT/p,p′-DDE ratios (3.3, 2.5, and 5, respectively) and concentrations of DDTs. In addition to the continental differences, land use was another significant driver of POP and PAH concentrations: vegetation from urban and industrial areas concentrated up to five orders of magnitude more POPs and PAHs than rural and remote areas (Fig. 2). Concentrations were similar or sometimes higher in rural than in remote areas. In contrast, differences among large plant types (e.g. lichens—plant-like composite organism, bryophytes, gymnosperms, and angiosperms) were less variable than concentrations among continents or land use areas at global scale (Fig. S2).

**Figure 3:**
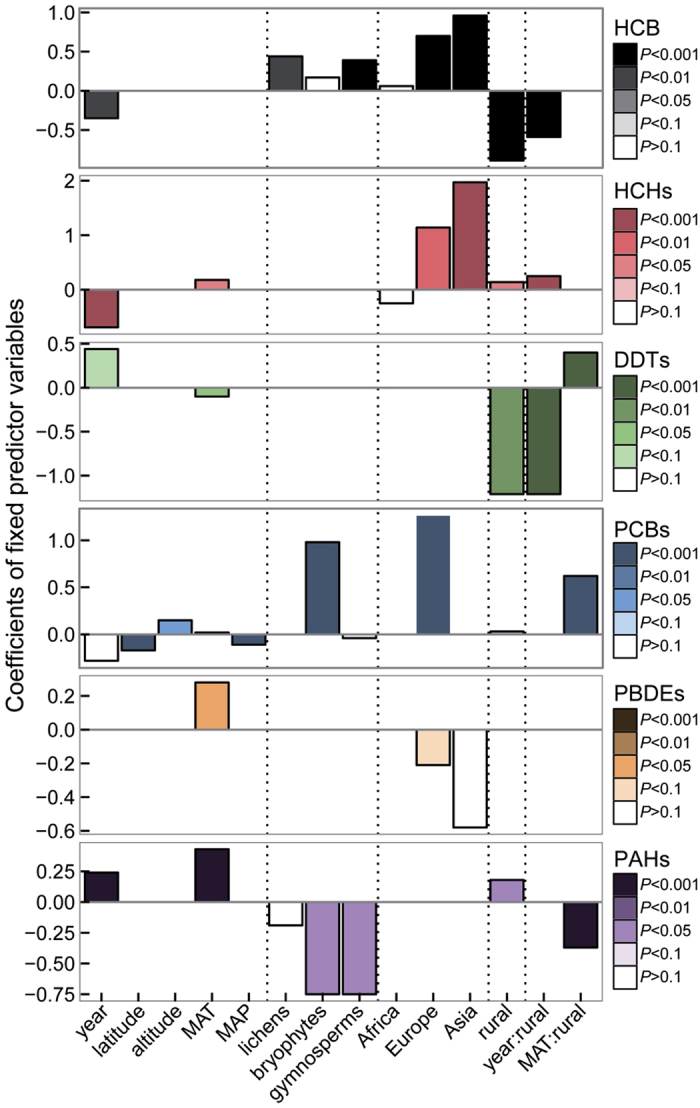
Multilevel model of the selected effects of abiotic and biotic variables on the concentrations of POPs in plants. Constant and varying effects by congeners are included for the predictor variables included in the lowest-AIC models. All models accounted for spatial correlation structure and nugget (which were significant in all cases, *P* < 0.001) and were run using the restricted maximum likelihood criterion for optimizing parameter estimates. MAT, mean annual temperature; MAP, mean annual precipitation.

We fit multilevel models (MLMs) to resolve the influences of location (continent or land use), plant type, climate, and temporal trends on the global distributions of POPs and PAHs in vegetation. The MLMs make it possible to estimate simultaneously the responses of total concentration of each family of compounds to predictor (independent) variables, in addition to also giving a summary of the predictor determinants of congener composition^21^. Totals for each family were always calculated based on the same, most usually measured compounds (see Methods for details). Year, latitude, altitude, mean annual temperature (MAT), mean annual precipitation (MAP), plant type, continent, and land use (urban and industrial study areas were excluded from the MLM to avoid potential point-source contamination) were included in the best-fitting multilevel models, selected using Akaike’s information criterion (AIC), as the main drivers determining the global distributions of POPs and PAHs in vegetation (Fig. 3, Table S3). Spatial autocorrelation was significant (*P* < 0.001) and included in all models.

**Figure 4:**
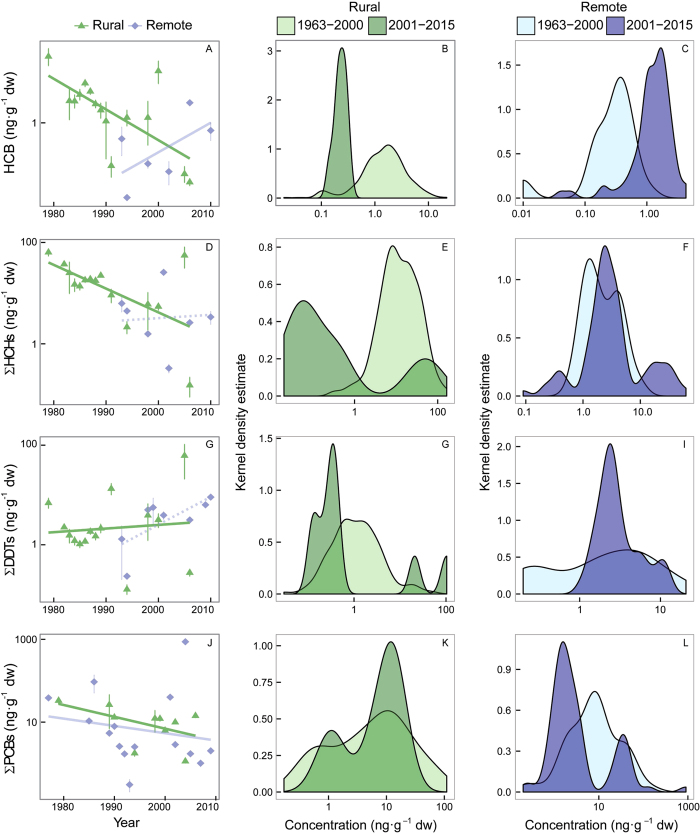
Individual relationships among HCB, ΣHCHs, and ΣDDTs (ng·g^−1^ dry weight) and year. The equations for rural and remote areas are: (**A**) HCB rural: y = −0.04× + 91, *r*^2^ = 0.19, df = 1354, *P* < 0.0001, and HCB remote: y = 0.07× − 140, *r*^2^ = 0.43, df = 1,95, *P* < 0.0001; (**D**) ΣHCHs rural: y = −0.07× + 148, *r*^2^ = 0.31, df = 1,356, *P* < 0.0001, and ΣHCHs remote: y = 0.08× − 16, *r*^2^ = 0.01, df = 1,143, *P* = 0.2; (**G**) ΣDDTs rural: y = −0.01× + 23, *r*^2^ = 0.01, df = 1,356, *P* = 0.09, and ΣDDTs remote: y = 0.01× − 13, *r*^2^ = 0.00, df = 1,155, *P* = 0.4; and (**J**) ΣPCBs rural: y = −0.02× + 50, *r*^2^ = 0.11, df = 1,102, *P* < 0.001, and ΣPCBs remote: y = −0.03× + 71, *r*^2^ = 0.24, df = 1,124, *P* < 0.0001. Panels (**B**), (**E**), (**H**) and (**K**) and (**C**), (**F**), (**I**) and (**L**) show the estimated curve for kernel density for both rural and remote regions, respectively, before and after 2000.

**Figure 5:**
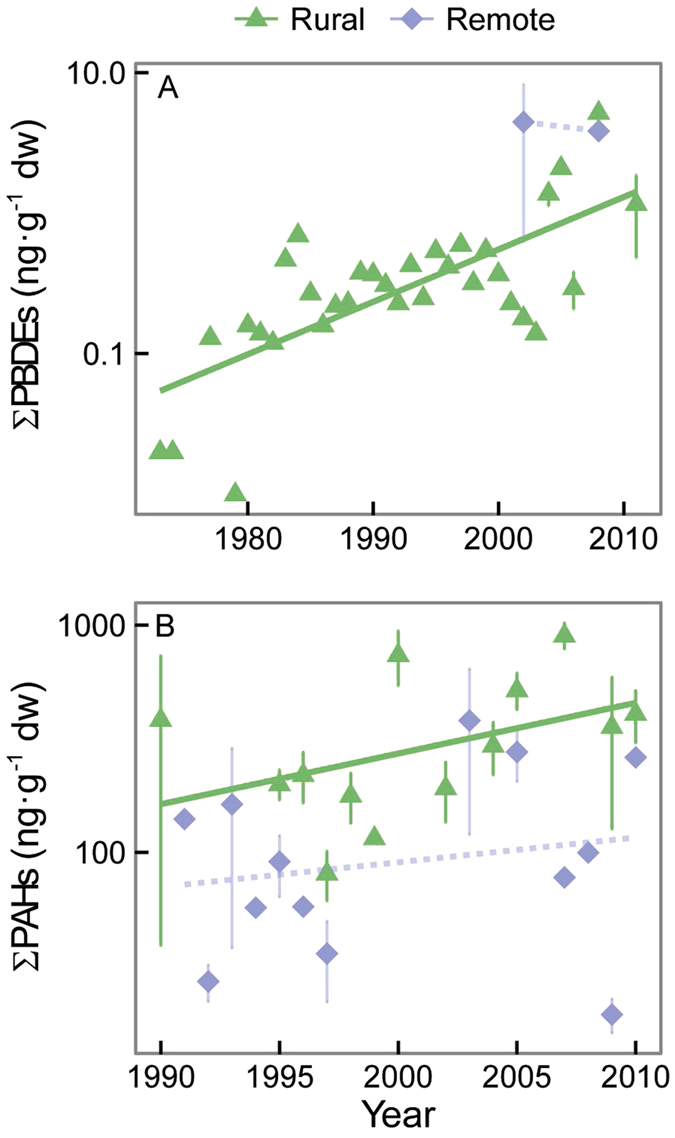
Individual relationships among ΣPBDEs and ΣPAHs (ng·g^−1^ dry weight) and year. The equations for rural and remote areas are: (**A**) ΣPBDEs rural: y = 0.05× − 98, *r*^2^ = 0.48, df = 1,91, *P* < 0.0001, and ΣPBDEs remote: y = 0.03× − 64, *r*^2^ = 0.05, df = 1,18, *P* = 0.3; and (**B**) ΣPAHs rural: y = 0.06× − 125, *r*^2^ = 0.32, df = 1,192, *P* < 0.0001, and ΣPAHs remote: y = −0.01× + 28, *r*^2^ = 0.02, df = 1,47, *P* = 0.3.

Continent and land use (remote and rural areas) were the strongest factors in most models accounting for the accumulation of POPs and PAHs in vegetation (Fig. 3). Year was also a significant variable explaining the accumulation of most POPs and PAHs in vegetation (Fig. 3). Year was associated negatively with the historically used HCB, HCHs, and PCBs, positively with the currently emitted PAHs, and not clearly associated with DDTs and PBDEs. Interestingly, this decreasing trend of the historically used compounds was not consistent among land-use areas, indicated by the positive interaction between year and land use (Fig. 3). Indeed, HCB, HCH, and DDT concentrations decreased only in rural areas but increased at remote sites (e.g. HCB, Fig. 4A) or showed no trend (e.g. HCHs and DDTs, Fig. 4D,G). In contrast, PBDE and PAH concentrations increased with time in rural areas and had no significant relationship with time in remote areas (Fig. 5, Fig. S3 for gymnosperms).

The strength of the climatic variables accounting for the variation of POPs and PAHs was smaller than all other variables, but still significant as expected at this scale. HCHs, PBDEs, and PAHs were positively associated with MAT, and PCBs were negatively associated with latitude and MAP (Fig. 3), indicating a dependence on fresh release from diffuse or point sources in warmer temperate areas. DDTs, however, were negatively associated with MAT, and PCBs were positively associated with altitude (Fig. 3), suggesting global fractionation^22^,^23^.

**Figure 6:**
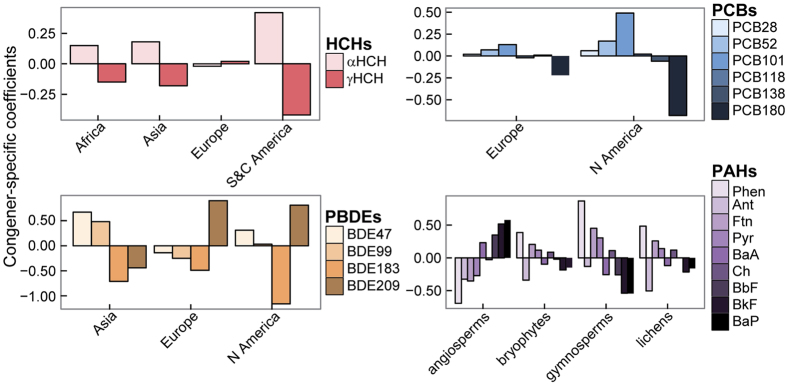
Coefficients from the multilevel model giving the significant congener-specific responses to continent and plant type*. The values are the species-specific changes of slope plus the estimates for fixed effects. *Lichens as plant-like composite organisms.

In the same MLMs, the different congeners of HCHs, PCBs, PBDEs, and PAHs had different concentration pattern depending on the continent or the plant type (different slope per congener in the MLM, Fig. 6). Concentrations were usually higher for α-HCH than γ-HCH on all continents except Europe. PCB101 was the PCB congener that bioaccumulated the most in vegetation, followed by lighter (PCB28 and 52) and heavier (PCB118, 138, and 180) PCBs. These differences were slightly more accentuated in North America. Concentrations were higher for BDE209 than all other PBDEs in North America and Europe, but were lower in Asia. The most important differences in PAH concentrations were in angiosperms versus gymnosperms, mosses and lichens, with higher proportions of PAHs of high molecular weight.

## Discussion

The global distribution of POPs and PAHs in vegetation is remarkable. The concentrations presented in this review generally did not exceed the thresholds established in toxicological assays^24^,^25^,^26^ but were nevertheless high considering the dates most of these compounds were restricted or banned. These concentrations may be of concern, because these hydrophobic and persistent compounds biomagnify across food webs, sometimes achieving toxic concentrations in top predators, including humans^27^.

Despite the high variability in POP and PAH sources and concentrations and in the number and location of studies that have analyzed these compounds in vegetation (Table S2), the industrialization history and distance to emission sources were key parameters determining the distribution of POPs and PAHs total concentration and congener composition around the globe, beyond plant type (with the different plant physiologies and life cycles compared) and climatic variables like temperature (Fig. 3). Europe was best represented for the number of studies and, together with Asia and North America, their vegetation accumulated the highest concentrations of most compounds, with the exception of HCHs and DDTs that dominated in remote areas of Africa, Asia, and Central America. These results concurred for example with soil global PCB budgets^28^ and PCB historical global production and consumption^29^. The proportion of the different congeners of HCHs, PCBs and PBDEs also differed among continents (Fig. 6). Concentrations were usually higher for α-HCH than γ-HCH everywhere except Europe, indicating a more direct and/or recent release of lindane (γ-) in Europe versus the historic HCH mixture known as technical HCH, which is less pure and has higher proportions of α-HCH^30^. Moderately hydrophobic PCB congeners (e.g. PCB101) dominated in most plant types, consistent with the McLachlan equilibrium model partitioning air and vegetation and considering the life span of the leaves and the effects of growth dilution and particle deposition^31^. PBDEs congener composition illustrated the stricter regulations in Europe and North America against the use and release of the less brominated PBDEs, while, Asia still presented higher proportion of these tetra- and penta-BDEs.

The concentrations of most compounds tended to decrease over time in rural areas, and those of other compounds have remained stable or have even increased at remote sites (Figs 3 and 4). The legislation established in most regions to reduce and eliminate the production, use, and release of most POPs thus appear to have been effective but insufficient, because POPs are still found and are accumulating in remote, usually cold, regions of the planet. Some studies at rural sites show a similar decreasing trend^16^,^32^, but less information is available for remote sites. These results seem thus to point to a problematic redistribution of POPs from point sources of contamination to remote regions. The travel of these semi-volatile compounds to remote regions where they have never been emitted depends on the physicochemical properties of the compounds and usually involves a cold condensation effect that traps these semivolatile compounds in colder areas^22^,^23^.

In rural areas, the concentrations of PAHs, PBDEs and DDTs did not experience the current decreasing trend observed for all other compounds reviewed here. In the case of PAHs, the trend of increasing PAH concentrations in vegetation over time is striking (Figs 3 and 5B). PAHs are emitted to the atmosphere from the burning of residential/commercial biomass (60.5%), open-field biomass (from agricultural waste, deforestation, and wildfires, 13.6%), and fossil fuels by on-road motor vehicles (12.8%)^33^. Jones *et al*.^34^ and Salamova *et al*.^35^ found evidence of a decline in PAH concentrations in rural vegetation in the UK and in the air at some sites around the Great Lakes in North America, but PAH concentrations in other locations show little evidence of decline (e.g. in air^35^,^36^ and mussels^37^) or even an increase (e.g. in air^36^ and mussels^38^). These variable temporal trends of PAHs concentrations, together with the positive relationship between PAHs concentrations and MAT in rural areas (Fig. 3), reinforce the strong influence of local/regional emission sources to PAH concentrations in the environment and underscore the concerns about the high PAH concentrations in the environment given the toxicity and environmental consequences of this group of compounds^39^. Unfortunately, with the dispersion of data we have, we could not find a strong enough signal of the different PAHs congeners in the MLM analysis to elucidate the sources (e.g. biogenic, industrial or degradative processes) of these compounds over time and/or across the different regions. However, we did find different congener patterns of PAHs among plant types (Fig. 6). Angiosperms accumulated higher proportions of high molecular weight PAHs in relation to other plant types. This pattern could be originated by a potential lower biotransformation capabilities of PAHs in angiosperms in relation to all other vegetation groups^40^. Similar results were reported for some arthropod lineages, which were more or less able to debrominate PBDEs depending on the evolutionary history of each organism^41^.

The concentrations of PBDEs similarly increased over time (Fig. 5A), but year was not selected as a predictor variable in the best MLM (Fig. 3), potentially due to the low sample sizes in past studies. Defining the global status of PBDEs is even more challenging than with other compounds due to the variation in PBDE regulations among countries. Finally, the global temporal increasing trend of DDT concentrations is mostly affected by the allowance of DDT use in regions with malaria and dengue fever to control populations of *Anopheles* mosquitoes^42^. Also, since dicofol (widely used as a pesticide in agriculture applications) is mainly synthesized from DDT, it contains DDT as an impurity (0.1–20%) and at present can therefore still be emitted to the atmosphere^43^. Indeed, Central America, Africa, and Asia had the highest DDT concentrations and very high p,p′-DDT/p,p′-DDE ratios, indicating recent use of DDT. Technical DDT generally contains 75% p,p′-DDT, 15% o,p′-DDT, 5% p,p′-DDE, and <5% other congeners, but p,p′-DDT is easily degraded in the environment to p,p′-DDE. Tanabe^44^ has identified the tropical belt as the major source of emission of organochlorine insecticides such as DDTs and HCHs, for the same reasons.

As expected at this global scale, the direct climatic influence on the distribution of POPs in vegetation was masked by the strong influence of development-derived factors such as continent, land use, or even year. The expected negative relationships of POPs concentrations along gradients of temperature (and latitude or altitude), consistent with the global distillation theory^22^,^23^, were only and slightly found for DDTs (MAT) and PCBs (altitude) (Fig. 3). In contrast, the positive effect of temperature on the distribution of POPs and PAHs around the globe, mostly in rural areas (significant interaction, Fig. 3), is likely underlining the different proximities to the sources of the sampling sites, which usually have higher temperatures^45^. These results reinforce the importance of not only the climatic and biogeochemical variables but also, and more importantly, other factors (such as proximity to sources, legislation, etc.) of each particular ecosystem on the overall multimedia partitioning of these persistent, hydrophobic, semi-volatile compounds at a global scale.

Strong efforts have been made to measure and assess the risk of particular POPs and PAHs in the global environment^29^,^46^,^47^, and similar efforts are starting to be developed for new chemicals such as current-use pesticides^48^. The constant release of new chemicals into the atmosphere every year, however, is large relative to the small fraction of chemicals measured^1^. Little is known in quantitative terms about the global sources of POP and PAH emissions. The implications for this progressive toxification of nature are still unresolved but certainly are one of the challenges that humanity will face in the near future. Our study demonstrates that (i) contamination levels in vegetation at rural and remote sites far from point sources of contamination are generally non-toxic but significant, and could cause adverse effects in the top trophic levels after being biomagnified, (ii) the accumulation of POPs and PAHs (and each particular congener) in vegetation depends on the industrialization history across continents and distance to emission sources, beyond plant type and climatic variables, such as temperature, and iii) global regulations initially reduced POP concentrations in vegetation in rural areas, but HCB, HCH, and DDT concentrations in remote sites are not decreasing and are even increasing (HCB) over time, suggesting a redistribution of these pollutants from their sources, while PBDE and PAH concentrations still show a hazardous increasing trend even in vegetation from rural areas. These concentrations found in vegetation consequently point to a concerning pollutant background in the majority of ecosystems of the planet that seems still far from being mitigated with the current international regulations. Changes in rates of transport and pools of POPs throughout the planet are additionally expected as the climate warms^49^, favoring re-emission of POPs from cold remote areas^50^,^51^ and increasing even more human and environmental exposure to POPs. Understanding the global load of these toxic persistent and semivolatile substances and the feedbacks between their dynamics and a changing environment is therefore crucial to enable the assessment and mitigation of this progressive toxification of nature.

## Methods

### Literature search and development of the database

We searched the Thomson Reuters Web of Knowledge database (http://thomsonreuters.com/) and Google Scholar (http://scholar.google.com/) for publications containing the *name of each compound, congener* or *group of compounds* and *vegetation*, or *plant/moss/lichen*, or *particular species of plant/moss/lichen*. We searched for additional studies by scanning the bibliographies of the publications identified by our initial search. Data were collected for 1977–2015 and 1432 locations around the globe (data summarized and references in Table S2). We extracted POP and PAH concentrations, plant/moss/lichen species names, locations, geographic coordinates, altitudes, and climatic variables for each location. All chemical concentrations were expressed in a dry weight basis (ng chemical per g dry weight). We did not express the concentrations in a lipid weight basis due to i) the low proportion of publications providing this information, ii) the low precision of the lipid gravimetric determination compared to total dry weight measurements, and iii) the presence of many other hydrophobic compounds such as membrane lipids and cuticular wax, cutin or terpenoids in addition to the lipids extracted by gravimetric methods^11^. Similarly, the possible influence of seasonal variation of starch content on the total dry weight basis^11^ was cushioned by the high number of samples considered and the assumption that the starch distribution was not skewed among sampling locations. Data from major urban or industrial centers were avoided and not used in the spatial analyses of this study.

Climatic or altitudinal information not available in the original articles were obtained from the WorldClim database 30 arc-second resolution grid^52^, and HadCRUT3 with 5° resolution grid^53^. The variables MAT, MAP, and altitude were selected from the WorldClim database, and cloud cover, potential evapotranspiration, and vapor pressure were selected from the HadCRUT3database. Antarctica was not well represented in these climatic databases, but most studies with data available from these polar locations fortunately also contained climatic data.

### Statistical analysis

All concentrations were logarithmically transformed to control for heteroscedasticity. Only needle and foliar concentrations were used for gymnosperms and angiosperms in the analyses. We used analysis of variance (ANOVA), Tukey’s honestly significant difference (HSD) test, and multivariate analysis of variance (MANOVA) to determine the variance of the concentrations of all congeners in each family of compounds among plant types and land uses. The ANOVA tested the differences among the sums of the concentrations for each family of compounds, and the MANOVA determined the variance of the concentrations of all congeners in each family of compounds.

We used MLMs^54^ to both simultaneously quantify the distributional patterns of POPs and PAHs in addition to the abundances of the constituent congeners^21^. The models can be interpreted as several regressions in which differences in slopes and intercepts among congeners are random variables. Using predictor (independent) variables enables this approach to assess the effects of abiotic and biotic drivers on POP and PAH concentrations and congener compositions while taking into account spatial correlation in the residuals. Without using predictor variables, this approach tests for spatial correlations in POP and PAH concentrations and congener compositions^55^. To do so, we only used the congeners better represented in the bibliography, which are for HCHs: α-HCH and γ-HCH, DDTs: DDT and DDE, PCBs: 28, 52, 101, 118, 138 and 180, PBDEs: 47, 99, 183 and 209, and PAHs: Phen, Ant, Ftn, Pyr, BaA, Ch, BbF, BkF and BaP.

The variables we tested were year of vegetation sampling, latitude, longitude, altitude, temperature, MAT, MAP, cloud cover, potential evapotranspiration, vapor pressure, and plant type (we grouped lichens (plant-like composite organism), bryophytes, gymnosperms, and angiosperms to simplify the analysis). All variables were transformed if needed to minimize skews. We excluded variables that were closely correlated to one another, so all abiotic and biotic variables listed above had pairwise correlations <0.7 (Cohen’s scale^56^). We checked for normality and homogeneity of residuals with Shapiro-Wilks and Levene’s tests. This analysis simultaneously provided information about abiotic and biotic drivers of the POP and PAH concentrations and the responses of individual congeners to these drivers^21^. Finally, spatial autocorrelation structure was significant in all analyses (*P* < 0.01) and was introduced in the model to correct for the spatial structure of the data.

Data were analyzed using R, v. 3.2.3^57^. Data from WorldClim and CRU were extracted using the R package *raster*, and the values of the four nearest raster cells were interpolated^58^. Data were analyzed using the R package *lme4*^59^. The maps were generated using the R packages *rgdal* (to project the latitude/longitude coordinates^60^)*, maptools* (to read in data from a shapefile and plotted the maps^61^), and *mapplots* (to add the pie charts to the maps^62^).

## Additional Information

**How to cite this article**: Bartrons, M. *et al*. Spatial And Temporal Trends Of Organic Pollutants In Vegetation From Remote And Rural Areas. *Sci. Rep*. **6**, 25446; doi: 10.1038/srep25446 (2016).

## Supplementary Material

Supplementary Information
