# Supplementary material for: Spatial And Temporal Trends Of Organic Pollutants In Vegetation From Remote And Rural Areas
Source: Sci Rep. 2016 May 5;6:25446. doi: 10.1038/srep25446 (PMC4857197; doi:10.1038/srep25446)
Supplement: Supplementary Information [file srep25446-s1.pdf]

SUPPLEMENTARY MATERIALS:

SPATIAL AND TEMPORAL TRENDS OF ORGANIC POLLUTANTS IN VEGETATION FROM REMOTE AND RURAL AREAS

Short title: Global distribution of POPs in vegetation

Authors: Mireia Bartrons,[1,2]* Jordi Catalan,[3] Josep Penuelas[1,3]

Affiliations:

[1] CSIC, Global Ecology Unit CREAF-CSIC-UAB. Cerdanyola del Vallès 08193, Barcelona, Catalonia, Spain.

[2] BETA Technological Centre (Tecnio), Aquatic Ecology Group, University of Vic–Central University of Catalonia. Vic 08500, Barcelona, Catalonia, Spain.

[3] CREAF. Cerdanyola del Vallès 08193, Barcelona, Catalonia, Spain.

*Corresponding author: mireia.bartrons@uvic.cat.

Table S1. Classification of the persistent organic pollutants (POPs) and polycyclic aromatic hydrocarbons (PAHs) in this study. Phen –Phenanthrene, Ant – Anthracene, Ftn – Fluoranthene, Pyr – Pyrene, BaA – Benz[a]anthracene, Ch – Chrysene, BbF – Benzo[b]fluoranthene, BkF – Benzo[k] fluoranthene, B[a]P – Benzo[a]pyrene.

**Use**		**POP**	**LogK_{OA}**	**LogK_{OW}**
Intentional	Fungicide and combustion byproduct of chlorinated organics	HCB	7.21	5.64
	Insecticides	HCHs		
		α-HCH	7.46	3.94
		γ-HCH	7.74	3.83
	Insecticides	DDTs		
		p,p'-DDE	9.70	4.20
		p-p'-DDT	9.73	6.39
	Industrial, dielectric/hydraulic fluid	PCBs		
		PCB28		5.71
		PCB52	7.82	5.79
		PCB101	8.24	6.40
		PCB118	9.82	6.57
		PCB138	9.06	6.70
		PCB153	9.55	6.80
		PCB180	10.20	7.21
	Industrial, flame retardant	PBDEs		
		BDE47	10.53	6.55
		BDE99	11.31	7.13
		BDE183	11.96	8.10
		BDE209	15.27	9.97
Unintentional	Forest fires and geochemical processes	PAHs		
		Phen	7.68	4.57
		Ant	7.71	4.45
		Ftn	8.76	5.22
		Pyr	8.93	5.18
		BaA	10.28	5.79
		Ch	10.3	5.73
		BbF	11.34	6.11
		BkF	11.37	6.11
		BaP	11.56	6.13

Sources: Harner and Shoeib [1], Tittlemier, et al. [2], Wania and Dugani [3], Xiao, et al. [4], Veltman, et al. [5], Mackay, et al. [6], Odabasi, et al. [7], and Daly, et al. [8], Finizio, et al. [9], Makino [10], Shiu and Ma [11]

Table S2. Plant types, common names, scientific names (when available), and POP concentrations (mean ± standard deviation in $ng \cdot g^{-1}$ dry weight) from the data presented in this study.

Group	Common name	Species	N	HCB	∑HCHs	∑DDTs	∑PCBs	∑PBDEs	∑PAHs
Lichen (plant-like composite organism)	lichen	*Lecanora aspidophora*, *Stereocaulon glabrum*, *Umbilicaria* spp., *Umbilicaria propagulifera*, *Usnea sulphurea*, *Hypogymnia physodes*, *Parmotrema hypoleucinum*, *Usnea* spp., *Usnea antarctica*, *Usnea aurantiaco-atra*	59	2.59 ± 3.36	42.73 ± 56.57	6.33 ± 7.98	28.86 ± 33.04	0.17 ± 0.06	641.7 ± 772.1
Bryophyta	moss	*Andreaea regularis*, *Bryum algens*, *Drepanocladus uncinatus*, *Brachytecyum* sp., *Hylocomium splendens*, *Pleurozium schreberi*, *Hypnum cupressiforme*, *H. plumaeformae*, *Isopterygium minutirameum*, *Sanionia uncinata*, *Sphagnum* spp., *Syntrichia princeps*	213	1.97 ± 3.92	15.41 ± 24.71	2.73 ± 3.75	19.80 ± 20.38	1.23 ± 1.41	761.2 ± 847.8
Lichen & moss			15	0.41 ± 0.39	10.74 ± 13.87	9.52 ± 16.46			
Gymnosperm	cedar	*Cedrus deodara*, *Juniperus virginiana*	30	1.16 ± 1.59	0.27 ± 0.23	2.88 ± 2.83	15.90 ± 12.68	2.79 ± 2.21	
	cypress	*Cupressus torulosa*	4	1.03 ± 0.02	3.34 ± 0.92	11.17 ± 0.96			
	larch	*Larix decidua*	5	0.61	0.34		2.18 ± 1.25		
	pine	*Pinus canariensis*, *P. densata*, *P. densiflora*, *P. halepensis*, *P. koraiensis*, *P. massoniana*, *P. tabulaeformis*, *P. maximartinezii*, *P. nigra*, *P. pinaster*, *P. pinea*, *P. strobus*, *P. sylvestris* (N = 388), *P. tabulaeformis*, *P. taeda*, *P. thunbergii*, *P. uncinata*, *P. virginiana*	727	1.82 ± 1.52	14.58 ± 15.94	2.72 ± 8.38	19.65 ± 32.91	267.7 ± 1035.3	542.3 ± 1120.8
	spruce	*Picea abies*, *Tsuga dumosa*	47	0.89 ± 0.50	3.03 ± 2.93	7.93 ± 11.91	31.58 ± 164.9	2.11	97.42 ± 123.3
Angiosperm	ash	*Fraxinus excelsior*, *Sorbus aucuparia*	13				1.71 ± 0.69		242.10
	beech	*Fagus sylvatica*	6	0.19	0.36		1.84 ± 1.17		
	camphor tree	*Cinnamomum camphora*	49					19.32 ± 57.24	
		Cinnamomum camphora, *Buxus* sp.	3					29.79 ± 24.31	
	chestnut	*Castanea sativa*	5	0.21	0.24		1.98 ± 0.94		
	elm	*Ulmus* spp.	3					57.45 ± 83.47	
	eucalyptus	*Eucalyptus* spp.	1					33.83	
	grass	*Deschampsia antarctica*, Poaceae	69	0.14 ± 0.08		0.08 ± 0.03	0.33 ± 0.30	1.55 ± 1.95	7.72 ± 2.86
	hazelnut	*Corylus avellana*	7	0.04	0.09		1.56 ± 0.72		577.5
	lime	*Tilia cordata*	4				1.71 ± 0.94		
	mango	*Mangifera indica*	10	0.11 ± 0.01	13.52 ± 37.58	24.30 ± 30.24			
	maple	*Acer pseudoplatanus*, *A. saccarum*	39	0.06	0.21		1.46 ± 0.90		766.7 ± 758.1

oak	*Quercus* spp., *Q. mongolica*, *Q. robur*	19					571.1 ± 661.8	283.20
poplar	*Populus*	1					101.5	
olive	*Olea europaea*	7	31 ± 16	91 ± 52	81 ± 63	255 ± 166		
corn	*Zea mays*	30						26.52 ± 11.85
oak, cherry tree, hornbeam, alderwood ashwood		1	0.33	81.20	103.90			
pearlwort	*Colobanthus quitensis*	3	0.53 ± 0.69	1.05	0.08 ± 0.04	7.31 ± 10.17	0.28	9.52

Sources: Lichen [12-21]; Bryophyta [13,15,17-19,21-39]; Lichen and moss [16]; Cedar [40,41]; Cypress [42]; Larch [43,44]; Pine [12,19,20,27,28,41,42,45-79]; Spruce [42-44,57,80-84]; Ash [43,85]; Beech [43,44]; Camphor tree [86,87]; Chestnut [43,44]; Elm [79]; Eucalyptus [68]; Grass [15,88,89]; Hazelnut [43,85]; Lime [43]; Mango [16]; Maple [43,44,72]; Oak [49,79,85]; Olive [90]; Poplar [79]; Corn [72]; Oak, cherry tree, hornbeam, alderwood and ashwood [36]; Pearlwort [15,18,21]

Table S3. Multilevel model of the effects of abiotic and biotic variables on the concentrations of POPs in plants. Constant and varying effects by congeners are included for the predictor variables included in the lowest-AIC models. All models accounted for spatial correlation structure and nugget (which were significant in all cases, $P < 0.001$) and were run using the restricted maximum likelihood criterion for optimizing parameter estimates. MAT, mean annual temperature; MAP, mean annual precipitation. •$P < 0.1$, *$P < 0.05$, **$P < 0.01$, ***$P < 0.001$

	**HCB (N = 419)**	**HCHs (N = 830)**		**DDTs (N = 754)**	**PCBs (N = 72)**		**PBDEs (N = 188)**		**PAHs (N = 648)**	
	**Fixed**	**Fixed**	**Random**	**Fixed**	**Fixed**	**Random**	**Fixed**	**Random**	**Fixed**	**Random**
**Year**	**-0.35*** **	**-0.70*** **		**0.44**	**-0.28*** **	**0.05***			**0.24*** **	
**Latitude**					**-0.17*** **					
**Altitude**					**0.15** **					
**MAT**		**0.18***		**-0.10***	**0.02**		**0.28***		**0.43*** **	
**MAP**					**-0.11*** **					
**Group, lichen**	**0.44***								**-0.19**	**0.33*** **
**Group, Bryophyta**	**0.17**				**0.98*** **				**-0.75***	**0.25*** **
**Group, gymnosperm**	**0.39** **				**-0.04**				**-0.75***	**0.48*** **
**Group, angiosperm**										
**Continent, North America**						**0.42*** **		**0.74*** **		
**Continent, Central & South America**			**0.43*** **							
**Continent, Europe**	**0.70*** **	**1.14***	**0.02*** **		**1.26*** **	**0.13*** **	**-0.21**	**0.54*** **		
**Continent, Africa**	**0.06**	**-0.25**	**0.15*** **							
**Continent, Asia**	**0.96*** **	**1.97*** **	**0.19*** **				**-0.58**	**0.61*** **		
**Land use, rural**	**-0.89*** **	**0.14***		**-1.21** **	**0.03**				**0.18***	
**Year:rural**	**-0.59*** **	**0.25*** **		**-1.21*** **						
**MAT:rural**				**0.40*** **	**0.62*** **				**-0.37*** **	

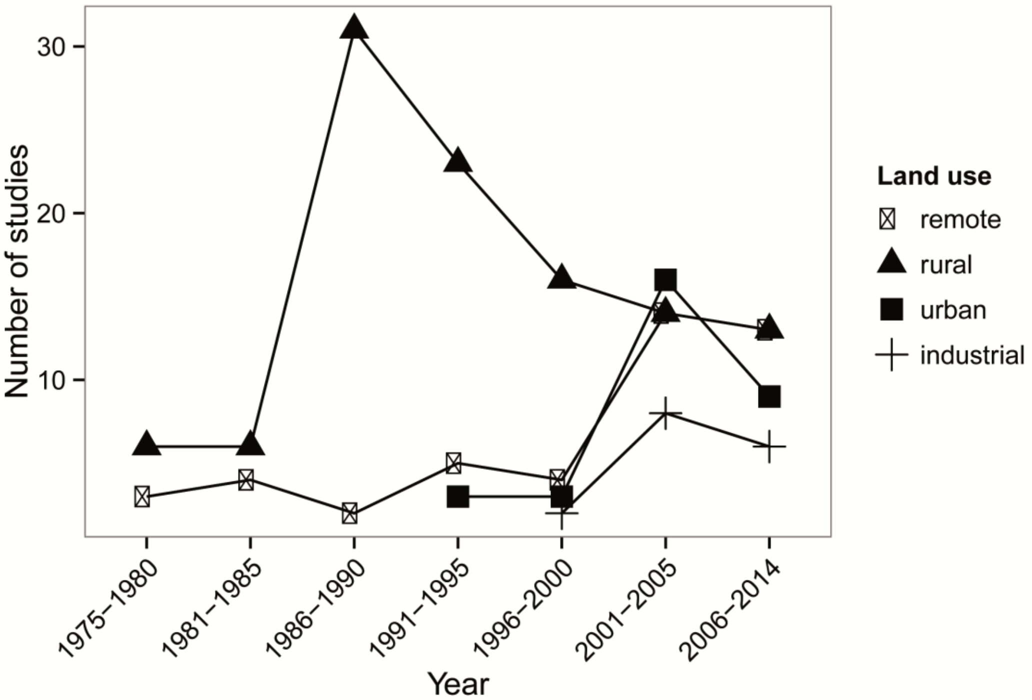

Fig. S1. Temporal evolution of the number of studies on POPs and PAHs in vegetation at remote, rural, urban, and industrial sites.

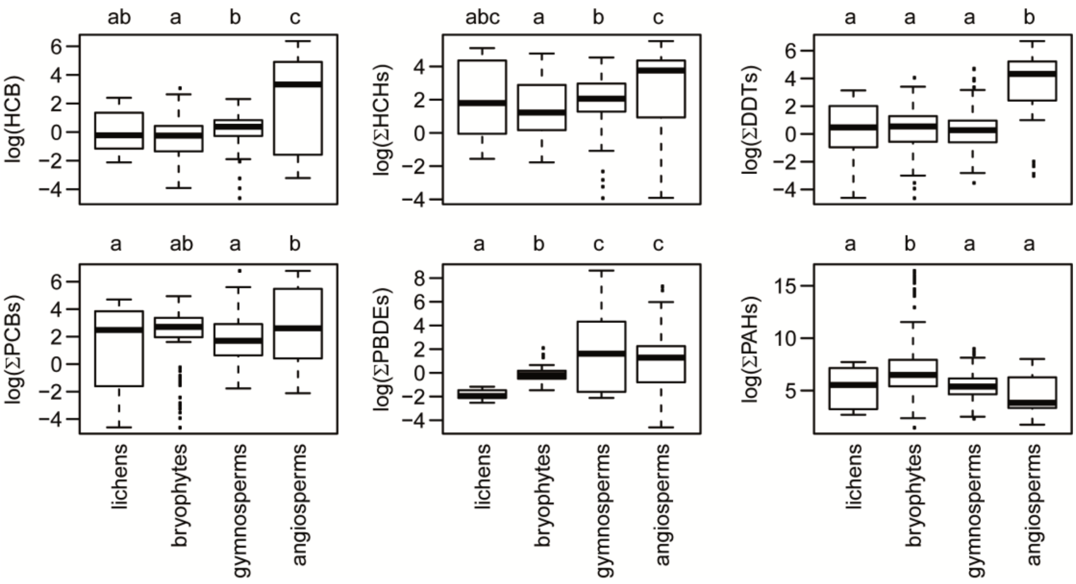

Fig. S2. Variability of the logarithm of the concentration of POPs and PAHs in each vegetation group. Different letters indicate significant differences among groups (Tukey's HSD, $P < 0.05$). Interquartile ranges (25th and 75th percentile) are shown by the height of the boxes, and the horizontal lines represent medians (50th percentile). Whiskers range from the 10th to 90th percentiles, and values outside this range are indicated by small squares. Concentrations are in $ng \cdot g^{-1}$ dry weight.

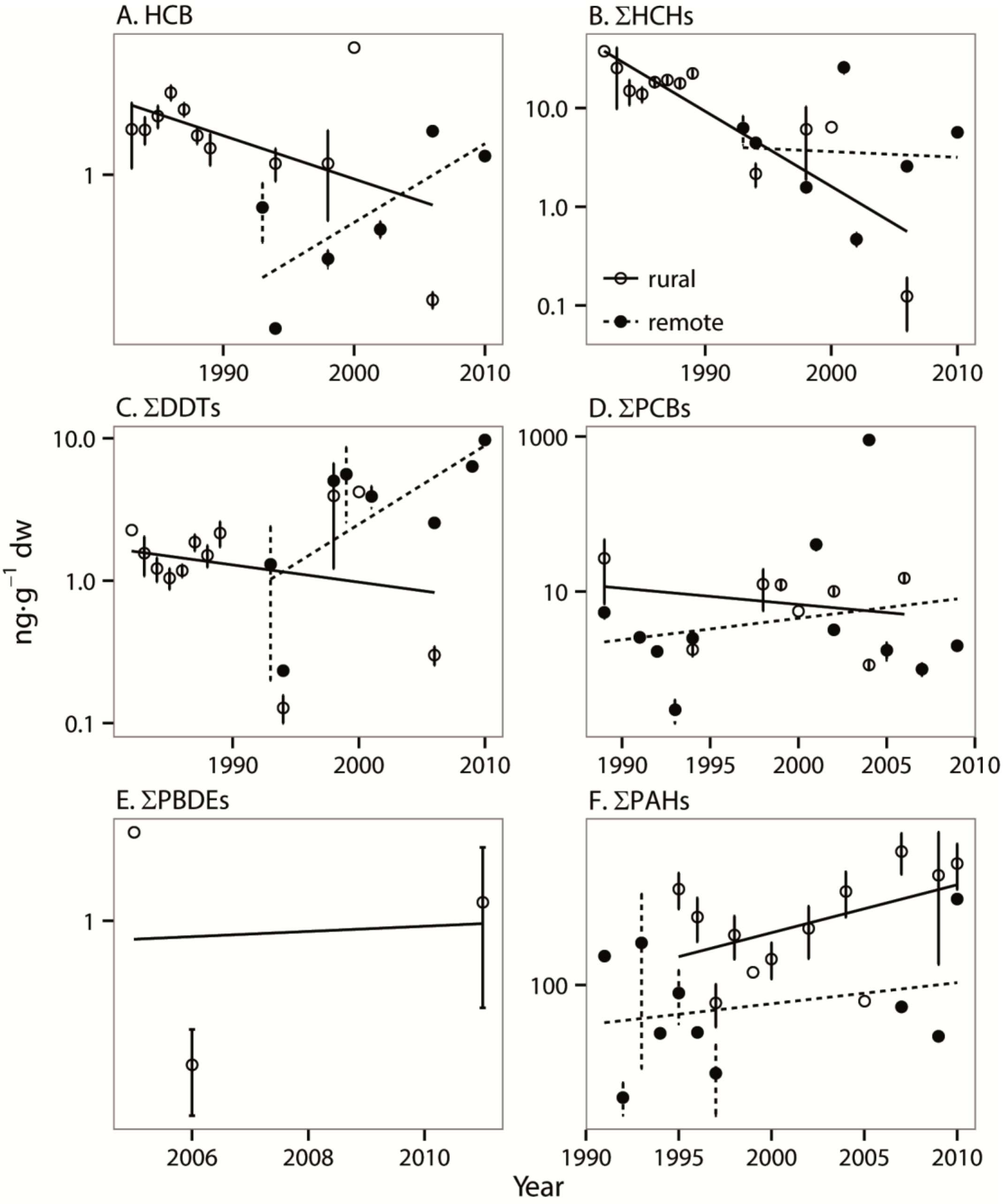

Fig. S3. Individual relationships among HCB, ΣHCHs, ΣDDTs, ΣPCBs, ΣPBDEs, and ΣPAHs (ng·g^{-1} dry weight) and year in gymnosperms. The equations for remote and rural areas are: A) HCB rural: y = -0.04x + 74, r^2 = 0.12, df = 1,303, P < 0.0001, and HCB remote: y = 0.08x - 152, r^2 = 0.56, df = 1,84, P < 0.0001; B) ΣHCHs rural: y = -0.08x + 171, r^2 = 0.33, df = 1,306, P < 0.0001, and ΣHCHs remote: y = -0.02x + 32, r^2 = 0.02, df = 1,101, P = 0.1; C) ΣDDTs rural: y = -0.02x + 40, r^2 = 0.02, df = 1,306, P = 0.009, and ΣDDTs rural: y = 0.03x - 58, r^2 = 0.10, df = 1,104, P < 0.001; D) ΣPCBs rural: y = 0.06x - 113, r^2 = 0.19, df = 1,77, P < 0.0001, and ΣPCBs remote: y = 0.02x - 31, r^2 = 0.02, df = 1,56, P = 0.3; E) ΣPBDEs rural: y = 0.05x - 98, r^2 = 0.06, df = 1,8, P = 0.5; and F) ΣPAHs rural: y = 0.03x - 55, r^2 = 0.11, df = 1,68, P = 0.005, and ΣPAHs remote: y = 0.02x - 38, r^2 = 0.14, df = 1,15, P = 0.1.

REFERENCES

1. Harner, T. & Shoeib, M. Measurements of octanol-air partition coefficients (K-OA) for polybrominated diphenyl ethers (PBDEs): Predicting partitioning in the environment. *Journal. Chem. Eng. Data.* **47**, 228-232 (2002).
2. Tittlemier, S. A., Halldorson, T., Stern, G. A. & Tomy, G. T. Vapor pressures, aqueous solubilities, and Henry's law constants of some brominated flame retardants. *Environ. Toxicol. Chem.* **21**, 1804-1810 (2002).
3. Wania, F. & Dugani, C. B. Assessing the long-range transport potential of polybrominated diphenyl ethers: A comparison of four multimedia models. *Environ. Toxicol. Chem.* **22**, 1252-1261 (2003).
4. Xiao, H., Li, N. Q. & Wania, F. Compilation, evaluation, and selection of physical-chemical property data for alpha-, beta-, and gamma-hexachlorocyclohexane. *Journal. Chem. Eng. Data.* **49**, 173-185, doi:10.1021/je034214i (2004).
5. Veltman, K. *et al.* Accumulation of organochlorines and brominated flame retardants in estuarine and marine food chains: Field measurements and model calculations. *Mar. Pollut. Bull.* **50**, 1085-1102 (2005).
6. Mackay, D., Shiu, W. Y., Ma, K. C. & Lee, S. C. *Handbook of Physical-Chemical Properties and Environmental Fate for Organic Chemicals, Second Edition*. (Taylor & Francis, 2006).
7. Odabasi, M., Cetin, E. & Sofuoglu, A. Determination of octanol-air partition coefficients and supercooled liquid vapor pressures of PAHs as a function of temperature: Application to gas-particle partitioning in an urban atmosphere. *Atmos. Environ.* **40**, 6615-6625, doi:10.1016/j.atmosenv.2006.05.051 (2006).
8. Daly, G. L., Lei, Y. D., Castillo, L. E., Muir, D. C. G. & Wania, F. Polycyclic aromatic hydrocarbons in Costa Rican air and soil: A tropical/temperate comparison. *Atmos. Environ.* **41**, 7339-7350, doi:10.1016/j.atmosenv.2007.05.014 (2007).
9. Finizio, A., Mackay, D., Bidleman, T. & Harner, T. Octanol-air partition coefficient as a predictor of partitioning of semi-volatile organic chemicals to aerosols. *Atmos. Environ.* **31**, 2289-2296, doi:http://dx.doi.org/10.1016/S1352-2310(97)00013-7 (1997).
10. Makino, M. Prediction of n-octanol/waterpartition coefficients of polychlorinated biphenyls by use of computer calculated molecular properties. *Chemosphere* **37**, 13-26, doi:http://dx.doi.org/10.1016/S0045-6535(98)00034-4 (1998).
11. Shiu, W.-Y. & Ma, K.-C. Temperature Dependence of Physical–Chemical Properties of Selected Chemicals of Environmental Interest. II. Chlorobenzenes, Polychlorinated Biphenyls, Polychlorinated Dibenzo-p-dioxins, and Dibenzofurans. *J. Phys. Chem. Ref. Data* **29**, 387-462, doi:doi:http://dx.doi.org/10.1063/1.1286267 (2000).
12. Augusto, S., Máguas, C., Matos, J., Pereira, M. J. & Branquinho, C. Lichens as an integrating tool for monitoring PAH atmospheric deposition: A comparison with soil, air and pine needles. *Environ. Pollut.* **158**, 483-489, doi:http://dx.doi.org/10.1016/j.envpol.2009.08.016 (2010).
13. Bacci, E. *et al.* Chlorinated hydrocarbons in lichen and moss samples from the antarctic peninsula. *Chemosphere* **15**, 747-754, doi:10.1016/0045-6535(86)90041-x (1986).
14. Blasco, M., Domeno, C., Lopez, P. & Nerin, C. Behaviour of different lichen species as biomonitors of air pollution by PAHs in natural ecosystems. *J Environ Monit* **13**, 2588-2596, doi:10.1039/c0em00681e (2011).

15 Cabrerizo, A., Dachs, J., Barceló, D. & Jones, K. C. Influence of Organic Matter Content and Human Activities on the Occurrence of Organic Pollutants in Antarctic Soils, Lichens, Grass, and Mosses. *Environ. Sci. Technol.* **46**, 1396-1405, doi:10.1021/es203425b (2012).

16 Calamari, D. *et al.* Role of plant biomass in the global environmental partitioning of chlorinated hydrocarbons. *Environ. Sci. Technol.* **25**, 1489-1495 (1991).

17 Carlberg, G. E., Ofstad, E. B., Drangsholt, H. & Steinnes, E. Atmospheric deposition of organic micropollutants in Norway studied by means of moss and lichen analysis. *Chemosphere* **12**, 341-356, doi:http://dx.doi.org/10.1016/0045-6535(83)90109-1 (1983).

18 Cipro, C. V. *et al.* Organic pollutants and their correlation with stable isotopes in vegetation from King George Island, Antarctica. *Chemosphere* **85**, 393-398, doi:10.1016/j.chemosphere.2011.07.047 (2011).

19 Migaszewski, Z. M., Galuszka, A. & Paslawski, P. Polynuclear aromatic hydrocarbons, phenols, and trace metals in selected soil profiles and plant bioindicators in the Holy Cross Mountains, south-central Poland. *Environ Int* **28**, 303-313 (2002).

20 Yang, R., Zhang, S., Li, A., Jiang, G. & Jing, C. Altitudinal and spatial signature of persistent organic pollutants in soil, lichen, conifer needles, and bark of the southeast Tibetan Plateau: implications for sources and environmental cycling. *Environ Sci Technol* **47**, 12736-12743, doi:10.1021/es403562x (2013).

21 Yogui, G. T., Sericano, J. L. & Montone, R. C. Accumulation of semivolatile organic compounds in Antarctic vegetation: A case study of polybrominated diphenyl ethers. *Sci. Total Environ.* **409**, 3902-3908, doi:http://dx.doi.org/10.1016/j.scitotenv.2011.06.010 (2011).

22 Borghini, F., Grimalt, J. O., Sanchez-Hernandez, J. C. & Bargagli, R. Organochlorine pollutants in soils and mosses from Victoria Land (Antarctica). *Chemosphere* **58**, 271-278, doi:10.1016/j.chemosphere.2004.07.025 (2005).

23 Foan, L. *et al.* Reconstructing historical trends of polycyclic aromatic hydrocarbon deposition in a remote area of Spain using herbarium moss material. *Atmos. Environ.* **44**, 3207-3214, doi:http://dx.doi.org/10.1016/j.atmosenv.2010.05.019 (2010).

24 Focardi, S., Gaggi, C., Chemello, G. & Bacci, E. Organochlorine residues in moss and lichen samples from two Antarctic areas. *Polar Record* **27**, 241-244, doi:doi:10.1017/S003224740001264X (1991).

25 Gałuszka, A. Distribution patterns of PAHs and trace elements in mosses Hylocomium splendens (Hedw.) B.S.G. and Pleurozium schreberi (Brid.) Mitt. from different forest communities: A case study, south-central Poland. *Chemosphere* **67**, 1415-1422, doi:http://dx.doi.org/10.1016/j.chemosphere.2006.10.010 (2007).

26 Himberg, K. K. & Pakarinen, P. Atmospheric PCB deposition in Finland during 1970s and 1980s on the basis of concentrations in ombrotrophic peat mosses (Sphagnum). *Chemosphere* **29**, 431-440, doi:http://dx.doi.org/10.1016/0045-6535(94)90431-6 (1994).

27 Holoubek, I. *et al.* The use of mosses and pine needles to detect persistent organic pollutants at local and regional scales. *Environ. Pollut.* **109**, 283-292, doi:http://dx.doi.org/10.1016/S0269-7491(99)00260-2 (2000).

28 Holoubek, I., Klanova, J., Jarkovsky, J., Kubik, V. & Helesic, J. Trends in background levels of persistent organic pollutants at Kosetice observatory, Czech

Republic.2) Part II. Aquatic and terrestrial environments 1996-2005. *J Environ Monit* **9**, 564-571, doi:10.1039/b701096f (2007).

29 Knulst, J., Westling, H. O. & Brorström-Lundén, E. Airborne organic micropollutant concentrations in mosses and humus as indicators for local versus long-range sources. *Environ. Monit. Assess.* **36**, 75-91, doi:10.1007/BF00546986 (1995).

30 Krommer, V., Zechmeister, H. G., Roder, I., Scharf, S. & Hanus-Illnar, A. Monitoring atmospheric pollutants in the biosphere reserve Wienerwald by a combined approach of biomonitoring methods and technical measurements. *Chemosphere* **67**, 1956-1966, doi:10.1016/j.chemosphere.2006.11.060 (2007).

31 Lead, W. A., Steinnes, E. & Jones, K. C. Atmospheric Deposition of PCBs to Moss (Hylocomium splendens) in Norway between 1977 and 1990. *Environ. Sci. Technol.* **30**, 524-530, doi:10.1021/es950275s (1996).

32 Lim, T. B., Xu, R., Tan, B. & Obbard, J. P. Persistent organic pollutants in moss as bioindicators of atmospheric pollution in Singapore. *Chemosphere* **64**, 596-602, doi:10.1016/j.chemosphere.2005.11.007 (2006).

33 Liu, X. *et al.* Compositional fractionation of polycyclic aromatic hydrocarbons (PAHs) in mosses (Hypnum plumaeformae WILS.) from the northern slope of Nanling Mountains, South China. *Atmos. Environ.* **39**, 5490-5499, doi:http://dx.doi.org/10.1016/j.atmosenv.2005.05.048 (2005).

34 Mariussen, E. *et al.* Spatial patterns of polybrominated diphenyl ethers (PBDEs) in mosses, herbivores and a carnivore from the Norwegian terrestrial biota. *Sci. Total Environ.* **404**, 162-170, doi:http://dx.doi.org/10.1016/j.scitotenv.2008.06.005 (2008).

35 Migaszewski, Z. M., Gałuszka, A., Crock, J. G., Lamothe, P. J. & Dołęgowska, S. Interspecies and interregional comparisons of the chemistry of PAHs and trace elements in mosses Hylocomium splendens (Hedw.) B.S.G. and Pleurozium schreberi (Brid.) Mitt. from Poland and Alaska. *Atmos. Environ.* **43**, 1464-1473, doi:http://dx.doi.org/10.1016/j.atmosenv.2008.11.035 (2009).

36 Tarcau, D., Cucu-Man, S., Boruvkova, J., Klanova, J. & Covaci, A. Organochlorine pesticides in soil, moss and tree-bark from North-Eastern Romania. *Sci. Total Environ.* **456–457**, 317-324, doi:http://dx.doi.org/10.1016/j.scitotenv.2013.03.103 (2013).

37 Wang, Q. *et al.* Determination of dioxin-like polychlorinated biphenyls in soil and moss from Fildes Peninsula, Antarctica. *Chin. Sci. Bull.* **57**, 992-996, doi:10.1007/s11434-011-4969-y (2012).

38 Zechmeister, H. G. *et al.* Pilot study on road traffic emissions (PAHs, heavy metals) measured by using mosses in a tunnel experiment in Vienna, Austria. *Environ Sci Pollut Res Int* **13**, 398-405 (2006).

39 Godzik, B., Szarek-Łukaszewska, G., Kapusta, P. & Stępień, K. PAHs Concentrations in Poland Using Moss Pleurozium Schreberi as Bioindicator. *pbj* **59**, 137, doi:10.2478/pbj-2014-0019 (2014).

40 Chen, J., Zhao, H., Gao, L., Henkelmann, B. & Schramm, K. W. Atmospheric PCDD/F and PCB levels implicated by pine (Cedrus deodara) needles at Dalian, China. *Environ Pollut* **144**, 510-515, doi:10.1016/j.envpol.2006.01.039 (2006).

41 Sajwan, K. S., Senthil Kumar, K., Kelley, S. & Loganathan, B. G. Deposition of organochlorine pesticides, PCBs (Aroclor 1268), and PBDEs in selected plant species from a Superfund Site at Brunswick, Georgia, USA. *Bull Environ Contam Toxicol* **82**, 444-449, doi:10.1007/s00128-009-9646-3 (2009).

42 Yang, R. Q., Yao, T. D., Xu, B. Q., Jiang, G. B. & Zheng, X. Y. Distribution of organochlorine pesticides (OCPs) in conifer needles in the southeast Tibetan Plateau. *Environ. Pollut.* **153**, 92-100, doi:10.1016/j.envpol.2007.07.033 (2008).

43 Nizzetto, L. *et al.* Accumulation parameters and seasonal trends for PCBs in temperate and boreal forest plant species. *Environ. Sci. Technol.* **42**, 5911-5916, doi:10.1021/es800217m (2008).

44 Nizzetto, L. *et al.* Accumulation of Persistent Organic Pollutants in Canopies of Different Forest Types: Role of Species Composition and Altitudinal-Temperature Gradient. *Environ. Sci. Technol.* **40**, 6580-6586, doi:10.1021/es0605523 (2006).

45 Grimalt, J. O. & van Drooge, B. L. Polychlorinated biphenyls in mountain pine (Pinus uncinata) needles from Central Pyrenean high mountains (Catalonia, Spain). *Ecotoxicol Environ Saf* **63**, 61-67, doi:10.1016/j.ecoenv.2005.06.005 (2006).

46 Hanari, N. *et al.* Dioxin-like compounds in pine needles around Tokyo Bay, Japan in 1999. *J. Environ. Monit.* **6**, 305-312, doi:10.1039/b311176h (2004).

47 Hellström, A., Kylin, H., Strachan, W. M. J. & Jensen, S. Distribution of some organochlorine compounds in pine needles from Central and Northern Europe. *Environ. Pollut.* **128**, 29-48, doi:http://dx.doi.org/10.1016/j.envpol.2003.08.033 (2004).

48 Hwang, H.-M., Wade, T. L. & Sericano, J. L. Concentrations and source characterization of polycyclic aromatic hydrocarbons in pine needles from Korea, Mexico, and United States. *Atmos. Environ.* **37**, 2259-2267, doi:http://dx.doi.org/10.1016/S1352-2310(03)00090-6 (2003).

49 Kannan, N., Hong, S., Yim, U. & Shim, W. Biomonitoring background levels of PCBs and PBDEs in Seoul metropolitan atmosphere for possible health effects. *Toxicol. Environ. Health. Sci.* **1**, 109-116, doi:10.1007/BF03216471 (2009).

50 Kim, B.-H., Lee, S.-J., Mun, S.-J. & Chang, Y.-S. A case study of dioxin monitoring in and around an industrial waste incinerator in Korea. *Chemosphere* **58**, 1589-1599, doi:http://dx.doi.org/10.1016/j.chemosphere.2004.10.041 (2005).

51 Kozul, D. & Romanic, S. H. Distribution of organochlorine compounds in pine needles collected in Zagreb. *Bull Environ Contam Toxicol* **81**, 339-342, doi:10.1007/s00128-008-9476-8 (2008).

52 Kylin, H., Grimvall, E. & Ostman, C. Environmental monitoring of polychlorinated-biphenyls using pine needles as passive samplers. *Environ. Sci. Technol.* **28**, 1320-1324, doi:10.1021/es00056a021 (1994).

53 Kylin, H., Hellström, A., Nordstrand, E. & Zaid, A. Organochlorine pollutants in Scots pine needles—biological and site related variation within a forest stand. *Chemosphere* **51**, 669-675, doi:http://dx.doi.org/10.1016/S0045-6535(03)00101-2 (2003).

54 Lehndorff, E. & Schwark, L. Biomonitoring airborne parent and alkylated three-ring PAHs in the Greater Cologne Conurbation I: Temporal accumulation patterns. *Environ. Pollut.* **157**, 1323-1331, doi:10.1016/j.envpol.2008.11.037 (2009).

55 Lehndorff, E. & Schwark, L. Biomonitoring of air quality in the Cologne Conurbation using pine needles as a passive sampler—Part II: polycyclic aromatic hydrocarbons (PAH). *Atmos. Environ.* **38**, 3793-3808, doi:http://dx.doi.org/10.1016/j.atmosenv.2004.03.065 (2004).

56 Loganathan, B. G., Seaford, K. D., Owen, D. A., Kumar, K. S. & Sajwan, K. S. Accumulation of Polychlorinated Biphenyls in Pine Needles Collected from Residential and Industrial Areas in Western Kentucky. *J. Ky. Acad. Sci.* **68**, 37-49, doi:10.3101/1098-7096(2007)68[37:AOPBIP]2.0.CO;2 (2007).

57 Moeckel, C., Nizzetto, L., Strandberg, B., Lindroth, A. & Jones, K. C. Air-Boreal Forest Transfer and Processing of Polychlorinated Biphenyls. *Environ. Sci. Technol.* **43**, 5282-5289, doi:10.1021/es803505u (2009).

58 Ockenden, W. A., Steinnes, E., Parker, C. & Jones, K. C. Observations on persistent organic pollutants in plants: Implications for their use as passive air samplers and for POP cycling. *Environ. Sci. Technol.* **32**, 2721-2726, doi:10.1021/es980150y (1998).

59 Ok, G. *et al.* Monitoring of air pollution by polychlorinated dibenzo-p-dioxins and polychlorinated dibenzofurans of pine needles in Korea. *Chemosphere* **46**, 1351-1357, doi:http://dx.doi.org/10.1016/S0045-6535(01)00261-2 (2002).

60 Piccardo, M. T. *et al.* Pinus nigra and Pinus pinaster needles as passive samplers of polycyclic aromatic hydrocarbons. *Environ. Pollut.* **133**, 293-301, doi:10.1016/j.envpol.2004.05.034 (2005).

61 Rappolder, M., Schroeter-Kermani, C., Schaedel, S., Waller, U. & Koerner, W. Temporal trends and spatial distribution of PCDD, PCDF, and PCB in pine and spruce shoots. *Chemosphere* **67**, 1887-1896, doi:10.1016/j.chemosphere.2006.05.079 (2007).

62 Ratola, N., Amigo, J. & Alves, A. Levels and Sources of PAHs in Selected Sites from Portugal: Biomonitoring with Pinus pinea and Pinus pinaster Needles. *Arch. Environ. Contam. Toxicol.* **58**, 631-647, doi:10.1007/s00244-009-9462-0 (2010).

63 Ratola, N., Alves, A., Santos, L. & Lacorte, S. Pine needles as passive bio-samplers to determine polybrominated diphenyl ethers. *Chemosphere* **85**, 247-252, doi:10.1016/j.chemosphere.2011.06.005 (2011).

64 Romanic, S. H. & Krauthacker, B. Distribution of persistent organochlorine compounds in one-year and two-year-old pine needles. *Bull. Environ. Contam. Toxicol.* **77**, 143-148, doi:10.1007/s00128-006-1043-6 (2006).

65 Romanic, S. H. & Krauthacker, B. Are pine needles bioindicators of air pollution? Comparison of organochlorine compound levels in pine needles and ambient air. *Arh Hig Rada Toksikol* **58**, 195-199, doi:10.2478/v10004-007-0012-8 (2007).

66 Salamova, A. & Hites, R. A. Brominated and Chlorinated Flame Retardants in Tree Bark from Around the Globe. *Environ. Sci. Technol.* **47**, 349-354, doi:10.1021/es303393z (2012).

67 Simonich, S. L. & Hites, R. A. Importance of vegetation in removing polycyclic aromatic hydrocarbons from the atmosphere. *Nature* **370**, 49-51 (1994).

68 Tian, M. *et al.* Plant Uptake of Atmospheric Brominated Flame Retardants at an E-Waste Site in Southern China. *Environ. Sci. Technol.* **46**, 2708-2714, doi:10.1021/es203669n (2012).

69 Tomashuk, T. A., Truong, T. M., Mantha, M. & McGowin, A. E. Atmospheric polycyclic aromatic hydrocarbon profiles and sources in pine needles and particulate matter in Dayton, Ohio, USA. *Atmos. Environ.* **51**, 196-202, doi:http://dx.doi.org/10.1016/j.atmosenv.2012.01.028 (2012).

70 Tremolada, P., Burnett, V., Calamari, D. & Jones, K. C. A study of the spatial distribution of PCBs in the UK atmosphere using pine needles. *Chemosphere* **32**, 2189-2203, doi:http://dx.doi.org/10.1016/0045-6535(96)00127-0 (1996).

71 Villa, S., Finizio, A., Diaz Diaz, R. & Vighi, M. Distribution of Organochlorine Pesticides in Pine Needles of an Oceanic Island: The Case of Tenerife (Canary Islands, Spain). *Water, Air, and Soil Pollution* **146**, 335-349, doi:10.1023/A:1023906306701 (2003).

72 Wagrowski, D. M. & Hites, R. A. Polycyclic Aromatic Hydrocarbon Accumulation in Urban, Suburban, and Rural Vegetation. *Environ. Sci. Technol.* **31**, 279-282, doi:10.1021/es960419i (1996).

73 Wenzel, K. D. *et al.* Immission patterns of airborne pollutants in Argentina and Germany .2. Biomonitoring of organochlorine compounds and polycyclic aromatics. *Chemosphere* **34**, 2505-2518, doi:10.1016/s0045-6535(97)00096-9 (1997).

74 de Wit, C. A., Alaee, M. & Muir, D. C. Levels and trends of brominated flame retardants in the Arctic. *Chemosphere* **64**, 209-233, doi:10.1016/j.chemosphere.2005.12.029 (2006).

75 Wyrzykowska, B. *et al.* Source determination of highly chlorinated biphenyl isomers in pine needles – Comparison to several PCB preparations. *Environ. Pollut.* **143**, 46-59, doi:http://dx.doi.org/10.1016/j.envpol.2005.11.018 (2006).

76 Wyrzykowska, B. *et al.* Polychlorinated biphenyls and -naphthalenes in pine needles and soil from Poland - Concentrations and patterns in view of long-term environmental monitoring. *Chemosphere* **67**, 1877-1886, doi:10.1016/j.chemosphere.2006.05.078 (2007).

77 Xu, D., Zhong, W., Deng, L., Chai, Z. & Mao, X. Levels of extractable organohalogens in pine needles in China. *Environ Sci Technol* **37**, 1-6 (2003).

78 Xu, D., Deng, L., Chai, Z. & Mao, X. Organohalogenated compounds in pine needles from Beijing city, China. *Chemosphere* **57**, 1343-1353, doi:10.1016/j.chemosphere.2004.09.016 (2004).

79 Zhu & Hites, R. A. Brominated Flame Retardants in Tree Bark from North America. *Environ. Sci. Technol.* **40**, 3711-3716, doi:10.1021/es060225v (2006).

80 Brorstrom-Lunden, E. & Lofgren, C. Atmospheric fluxes of persistent semivolatile organic pollutants to a forest ecological system at the Swedish west coast and accumulation in spruce needles. *Environ. Pollut.* **102**, 139-149, doi:10.1016/s0269-7491(98)00081-5 (1998).

81 Di Guardo, A. *et al.* Conifer needles as passive biomonitors of the spatial and temporal distribution of DDT from a point source. *Chemosphere* **52**, 789-797, doi:10.1016/s0045-6535(03)00256-x (2003).

82 Offenthaler, I. *et al.* PCDD/F and PCB in spruce forests of the Alps. *Environ. Pollut.* **157**, 3280-3289, doi:http://dx.doi.org/10.1016/j.envpol.2009.05.052 (2009).

83 St-Amand, A. D., Mayer, P. M. & Blais, J. M. Seasonal trends in vegetation and atmospheric concentrations of PAHs and PBDEs near a sanitary landfill. *Atmos. Environ.* **42**, 2948-2958, doi:http://dx.doi.org/10.1016/j.atmosenv.2007.12.050 (2008).

84 Weiss, P., Lorbeer, G. & Scharf, S. Regional aspects and statistical characterisation of the load with semivolatile organic compounds at remote Austrian forest sites. *Chemosphere* **40**, 1159-1171, doi:http://dx.doi.org/10.1016/S0045-6535(99)00365-3 (2000).

85 Howsam, M., Jones, K. C. & Ineson, P. PAHs associated with the leaves of three deciduous tree species. I — Concentrations and profiles. *Environ. Pollut.* **108**, 413-424, doi:http://dx.doi.org/10.1016/S0269-7491(99)00195-5 (2000).

86 Ma, J. *et al.* Polybrominated dibenzo-p-dioxins/ dibenzofurans and polybrominated diphenyl ethers in soil, vegetation, workshop-floor dust, and electronic shredder residue from an electronic waste recycling facility and in soils from a chemical industrial complex in eastern China. *Environ Sci Technol* **43**, 7350-7356 (2009).

87 Zhao, Y. X. *et al.* Diffusion of polybrominated diphenyl ether (PBDE) from an e-waste recycling area to the surrounding regions in Southeast China. *Chemosphere* **76**, 1470-1476, doi:10.1016/j.chemosphere.2009.07.023 (2009).

88 Hassanin, A., Johnston, A. E., Thomas, G. O. & Jones, K. C. Time Trends of Atmospheric PBDEs Inferred from Archived U.K. Herbage. *Environ. Sci. Technol.* **39**, 2436-2441, doi:10.1021/es0486162 (2005).

89 Parolini, M., Guazzoni, N., Binelli, A. & Tremolada, P. Polybrominated Diphenyl Ether Contamination in Soil, Vegetation, and Cow Milk From a High-Mountain Pasture in the Italian Alps. *Arch. Environ. Contam. Toxicol.* **63**, 29-44, doi:10.1007/s00244-012-9753-8 (2012).

90 Gari, M., Bosch, C., Grimalt, J. O. & Sunyer, J. Impacts of atmospheric chlor-alkali factory emissions in surrounding populations. *Environ Int* **65**, 1-8, doi:10.1016/j.envint.2013.12.008 (2014).
